# Supplementary material for: Extremely Low-Frequency Electromagnetic Field (ELF-EMF) Increases Mitochondrial Electron Transport Chain Activities and Ameliorates Depressive Behaviors in Mice
Source: Int J Mol Sci. 2024 Oct 21;25(20):11315. doi: 10.3390/ijms252011315 (PMC11508854; doi:10.3390/ijms252011315)
Supplement: Supplementary file 1 [file ijms-25-11315-s001.zip › Supplementary Information.pdf]

Supplementary Materials for

Extremely low-frequency electromagnetic field (ELF-EMF) increases mitochondrial electron transport chain activities and ameliorates depressive behaviors in mice

Masaki Teranishi [1], Mikako Ito [1,*], Zhizhou Huang [1], Yuki Nishiyama [1], Akio Masuda [1], Hiroyuki Mino [2], Masako Tachibana [3], Toshiya Inada [4] and Kinji Ohno [1,5,*]

[1]Division of Neurogenetics, Center for Neurological Diseases and Cancer, Nagoya University Graduate School of Medicine, Nagoya, Japan

[2]Division of Material Science, Nagoya University Graduate School of Science, Nagoya, Japan

[3]Department of Psychiatry, Nagoya University Hospital, Nagoya, Japan

[4]Department of Psychiatry, Nagoya University Graduate School of Medicine, Nagoya, Japan

[5]Graduate School of Nutritional Sciences, Nagoya University of Arts and Sciences, Nisshin, Japan

This PDF file includes:

Supplementary Table S1

Supplementary Figure S1-S4

Supplementary Table S1. Antibodies for immunoblotting

Molecule	Incubation solution	Dilution	Supplier	Catalog number
β-actin	TBS-T	1:2000	Santa Cruz	sc-47778
VDAC1	TBS-T	1:1000	Abcam	ab14734
OXPHOS cocktail	TBS-T	1:1000	Abcam	ab110413
SDHA	TBS-T	1:1000	GeneTex	GTX101689
UQCRFS1	TBS-T	1:2000	Abcam	ab14746
Sirt3	Bullet Blocking One	1:500	Cell Signaling	cat#5490
FoxO3a	TBS-T	1:1000	Cell Signaling	cat#12829
SOD2	TBS-T	1:400	Santa Cruz	sc-30080
Ace-SOD2	Canget 1	1:500	Abcam	ab137037
4-HNE	TBS-T	1:500	JaICA	MHN-020P
Phospho-DRP1 (Ser616)	TBS-T	1:1000	Cell Signaling	cat#3455
DRP1	TBS-T	1:1000	Cell Signaling	cat#5391
OPA1	TBS-T	1:1000	Proteintech	cat#27733-1-AP
MFN1	TBS-T	1:1000	Proteintech	cat#13798-1-AP
PINK1	Canget 1	1:500	Abcam	ab23707
LC3-B	TBS-T	1:1000	Abcam	ab51520
Anti-mouse IgG, HRP-linked	TBS-T-Milk (5%)	1:2000	Cell Signaling	cat#7076
Anti-rabbit IgG, HRP-linked	TBS-T-Milk (5%) or Canget 2	1:2000	Cell Signaling	cat#7074

Canget 1, Can Get Signal Immunoreaction Enhancer Solution 1 for primary antibody (TOYOBO, NKB-101); Canget 2, Can Get Signal Immunoreaction Enhancer Solution 2 for secondary antibody (TOYOBO, NKB-301); Bullet Blocking One, Bullet Blocking One for Western Blotting (Nacalai Tesque, cat#13779-14); Milk, Blotto non-fat dry milk (Santa Cruz Biotechnology, sc-2325); and JaICA, Japan Institute for the Control of Aging.

Supplementary Figure S1

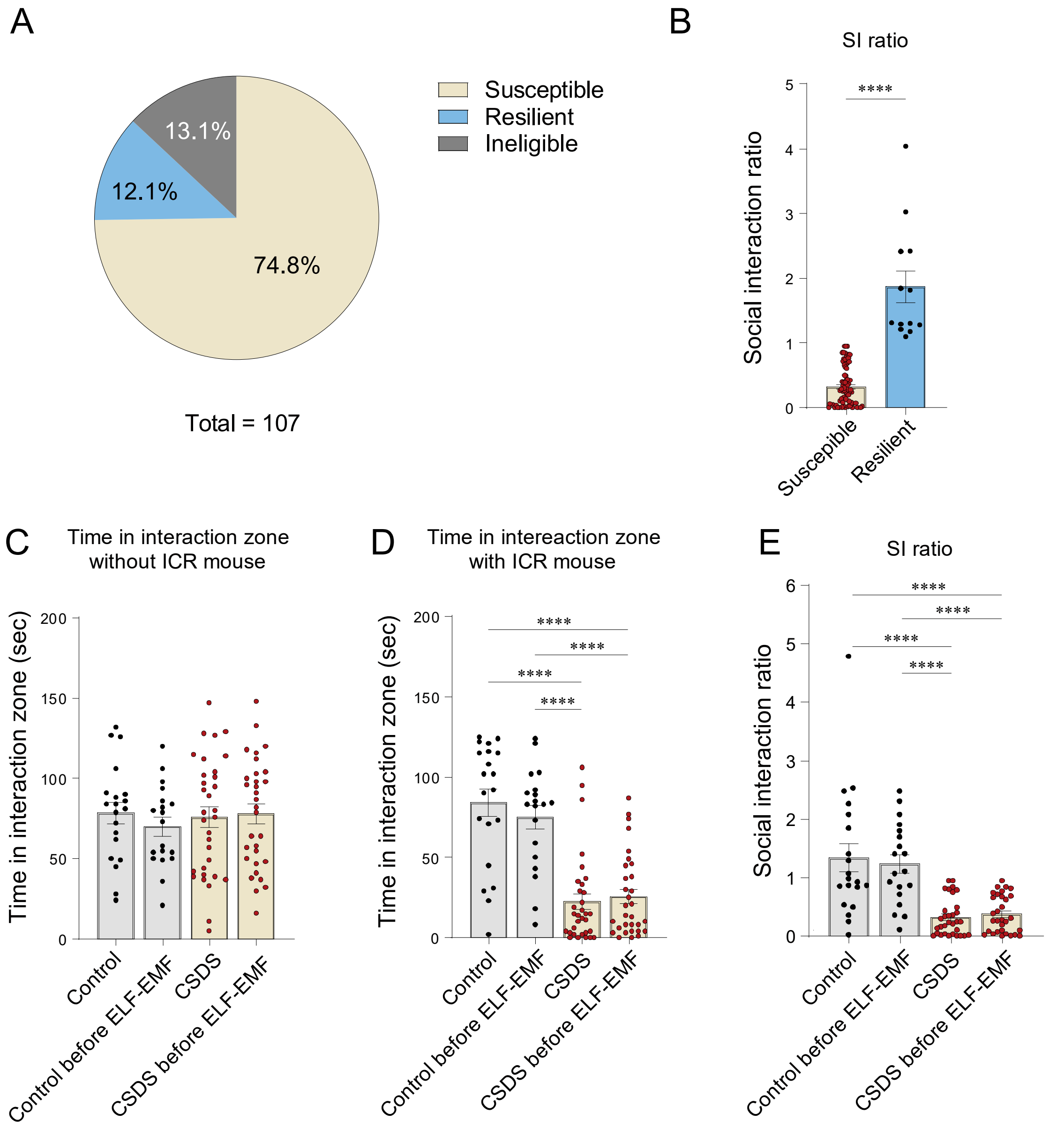

Supplementary Figure S1. Social interaction test on the next day after CSDS for 10 consecutive days to divide mice into four groups: two groups not to be exposed to ELF-EMF (Control and CSDS mice) and two groups to be exposed to ELF-EMF (before ELF-EMF and CSDS + before ELF-EMF mice). (A) Pie chart showing the percentages of susceptible (SI ratio < 1.0, $n = 80$), resilient (SI ratio ≥ 1.0, $n = 13$), and ineligible (deceased in the course of CSDS, $n = 14$). **(B)** SI ratios of susceptible and resilient CSDS-applied mice. $^{****}p < 0.0001$ by Student's *t*-test. **(C, D, E)** Time in the interaction zone without **(C)** and with **(D)** ICR mouse, and the SI ratio **(E)**. Note that Control and Control before ELF-EMF mice, as well as CSDS and CSDS before ELF-EMF mice, were divided so that both groups have similar SI ratios. Mean and SEM are indicated ($n = 19$ to 32 mice each). $^{****}p <$ 0.0001 by one-way ANOVA followed by Tukey's multiple comparisons test.

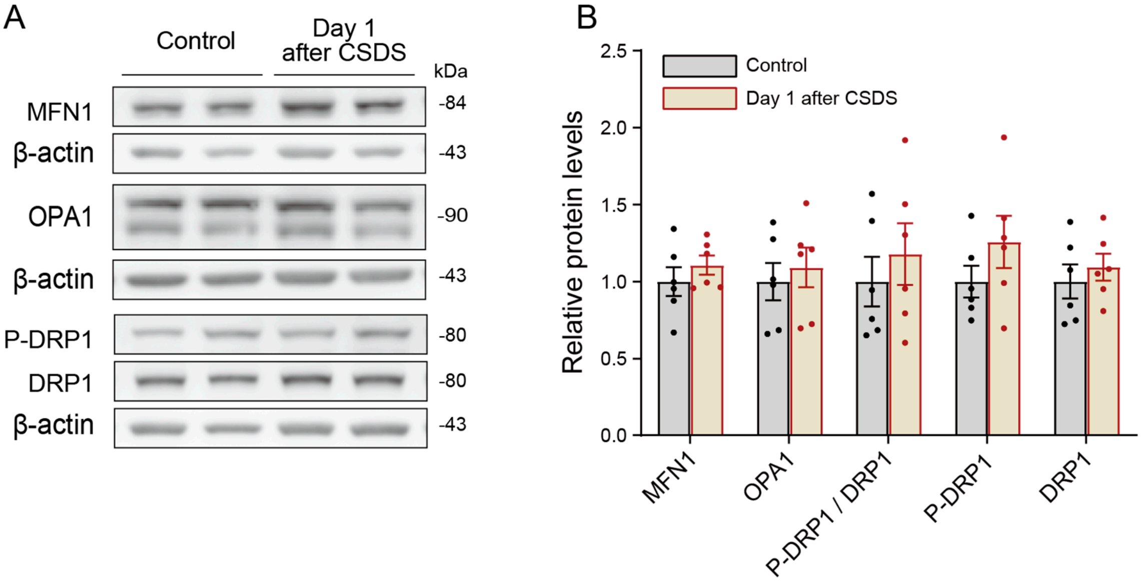

Supplementary Figure S2. CSDS for 10 consecutive days had no effects on the protein expressions of mitochondrial fusion markers, MFN1 and OPA1, or on the phosphorylation of a mitochondrial fission marker, DRP1. (A) Representative duplicated immunoblots on the next day after CSDS. **(B)** Densitometric analysis of immunoblots. Mean and SEM are indicated (n = 6 brain hemispheres each). No statistical difference by two-way ANOVA.

Supplementary Figure S3

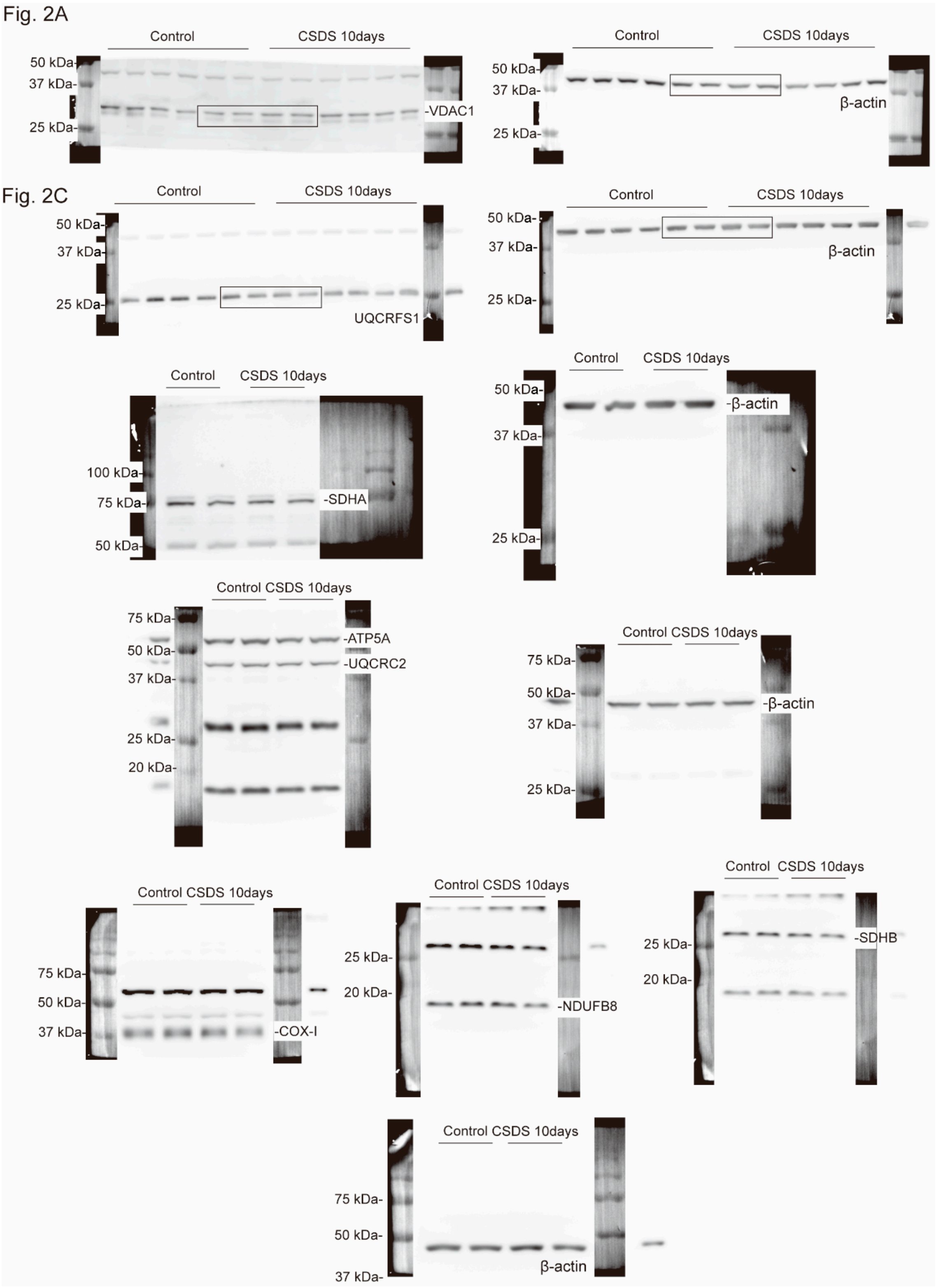

Supplementary Figure S3

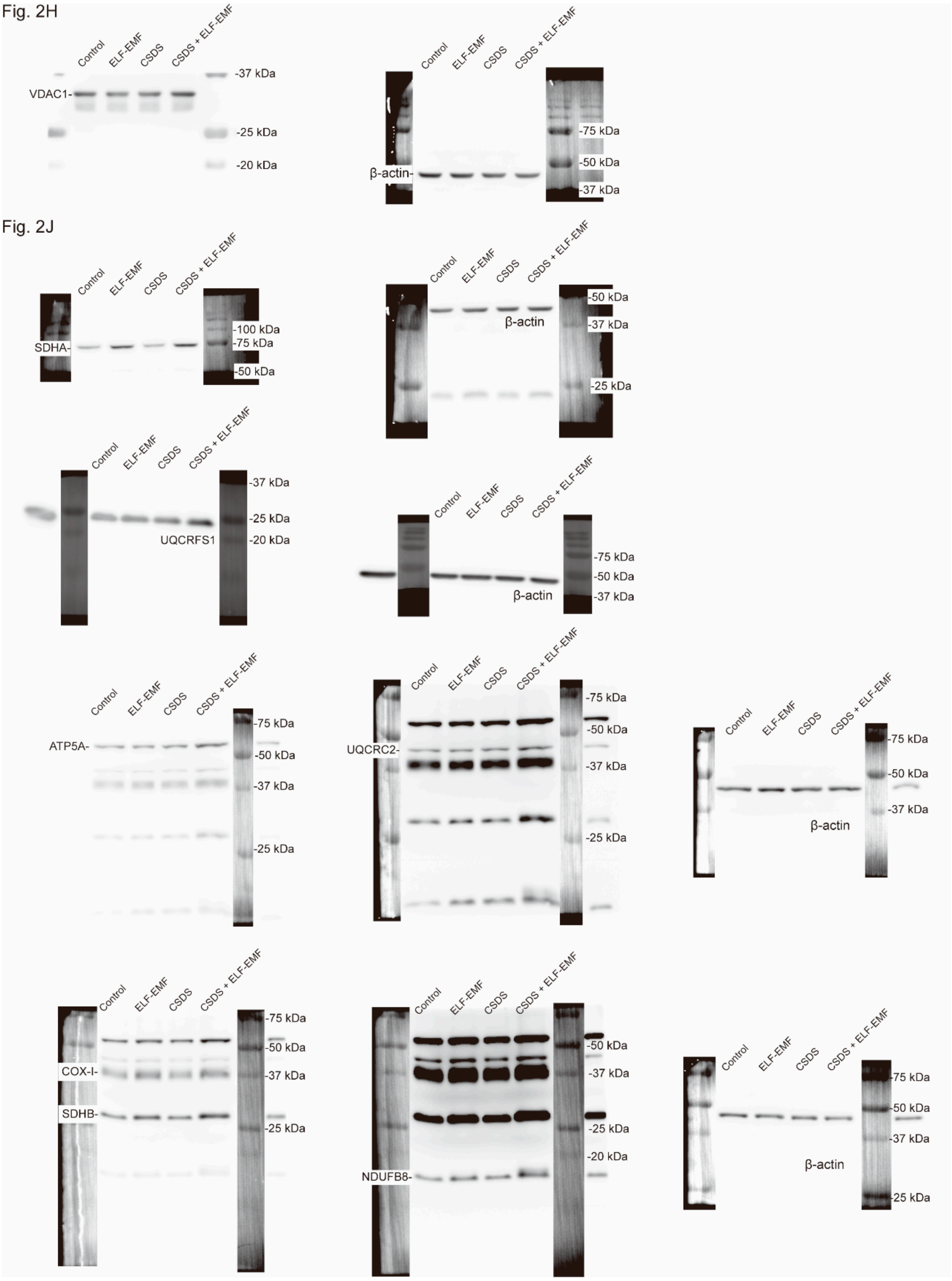

Supplementary Figure S3

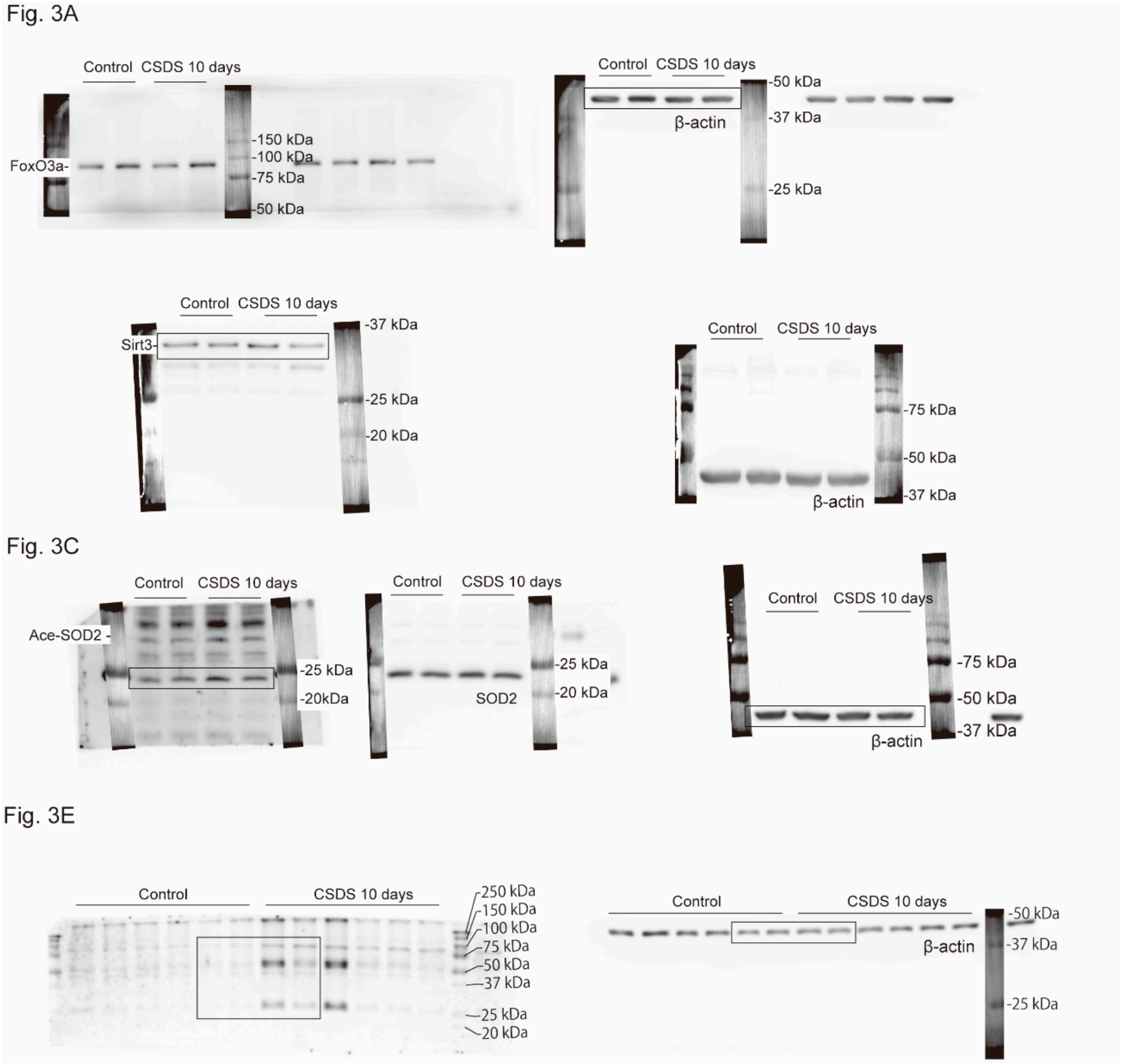

Supplementary Figure S3

Fig. 3G

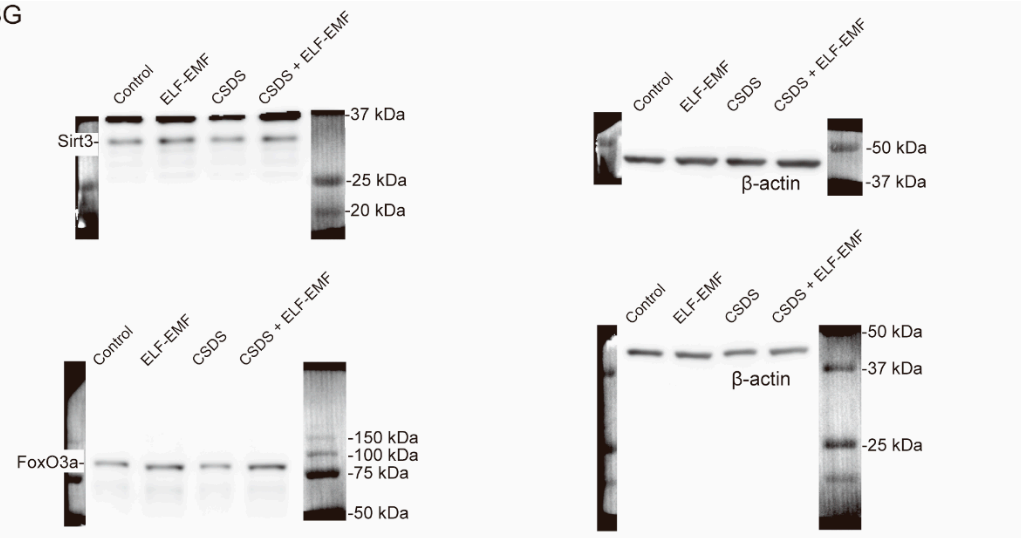

Fig. 3I

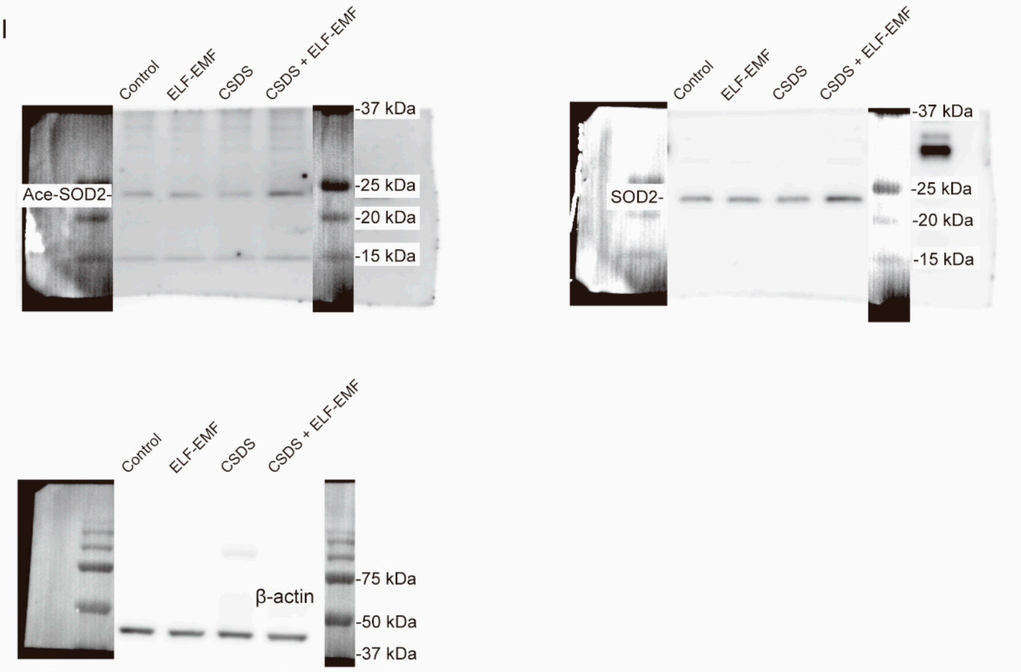

Fig. 3K

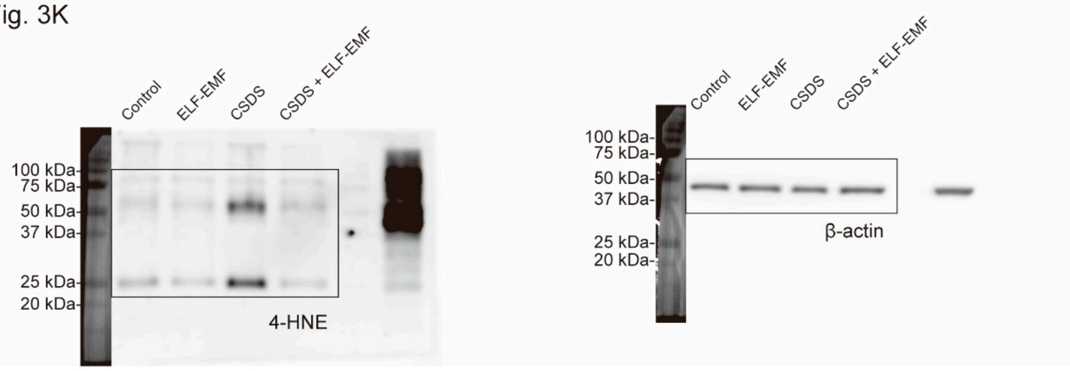

Supplementary Figure S3

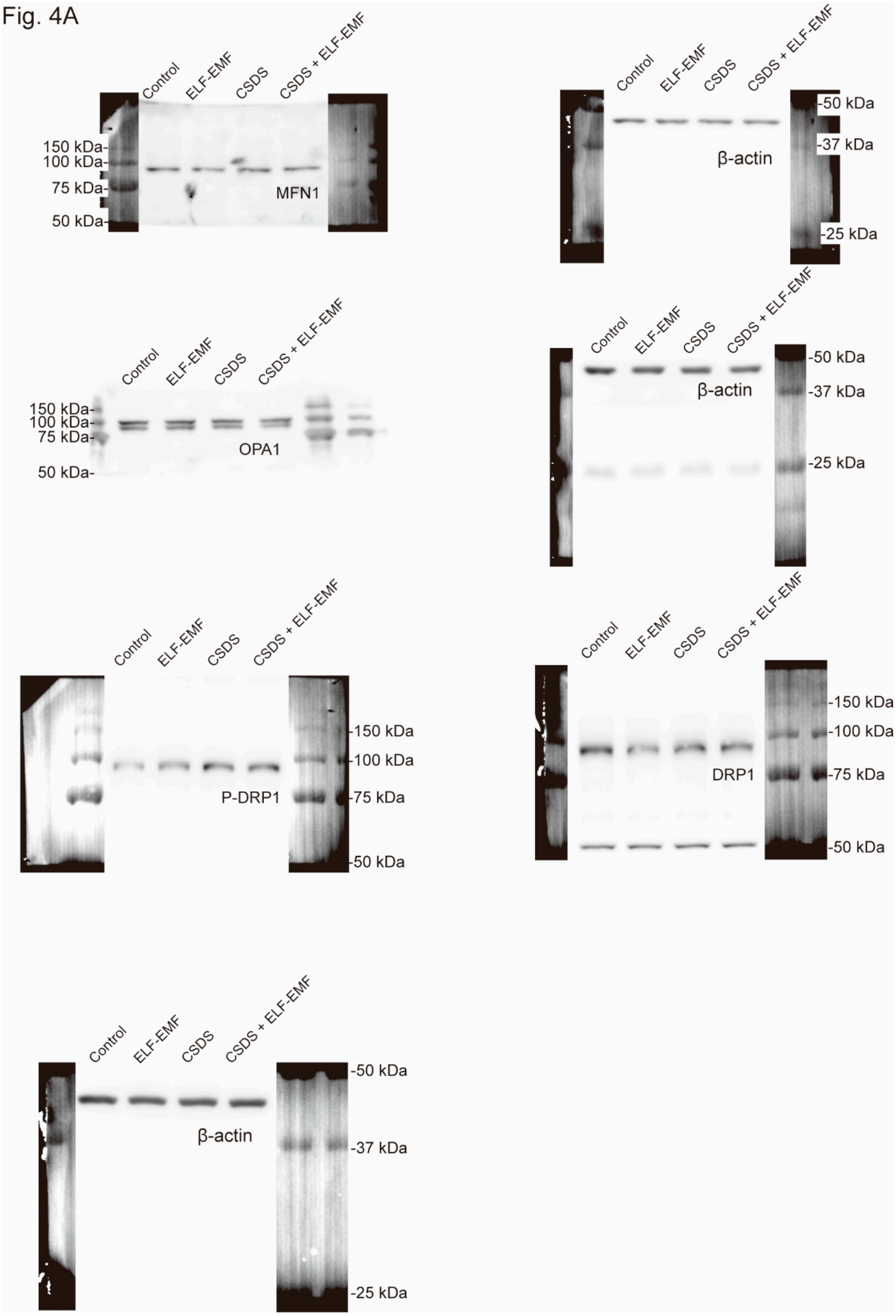

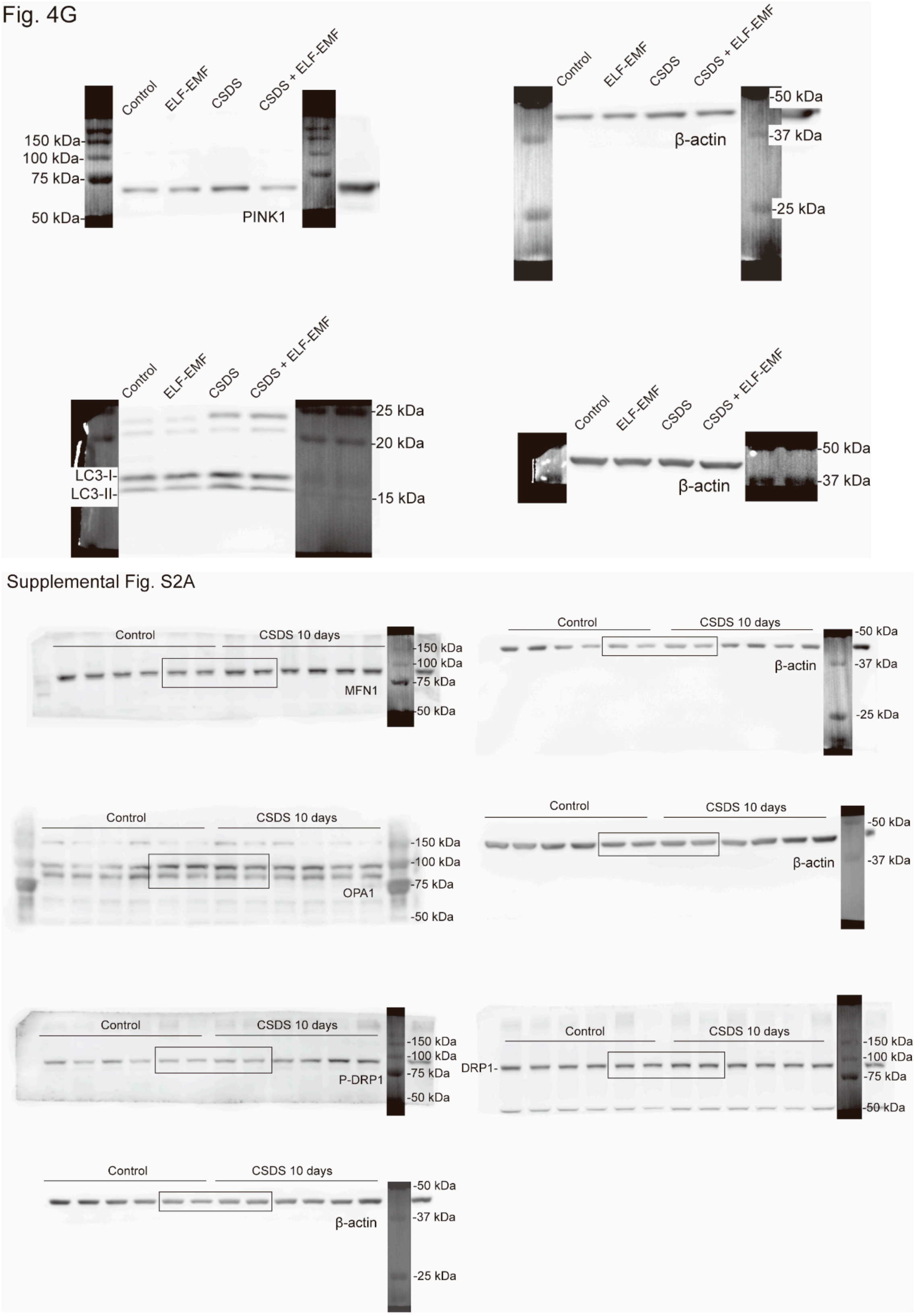

Supplementary Figure S3. Original immunoblots used as representative images

Supplementary Figure S4.

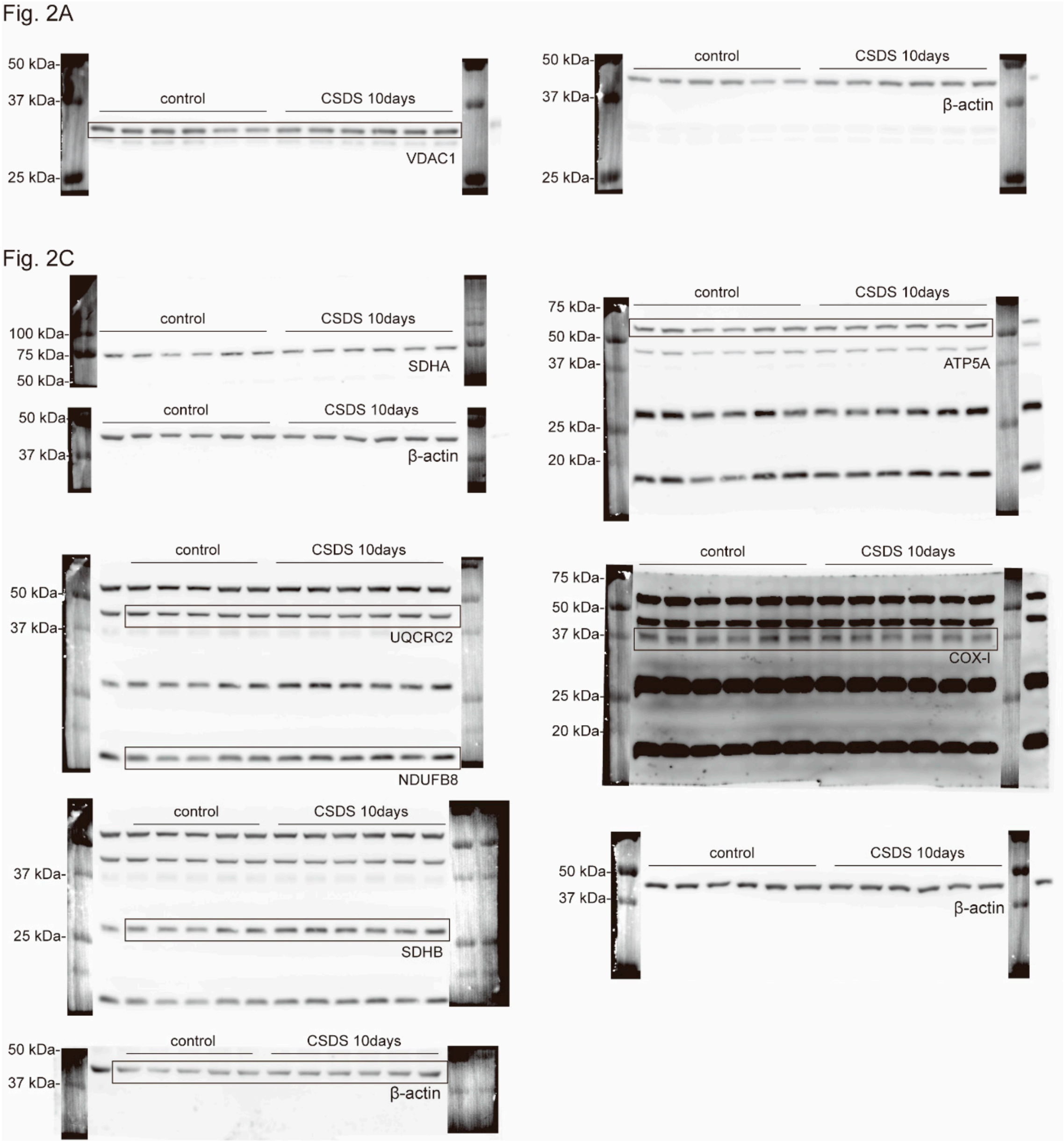

Supplementary Figure S4

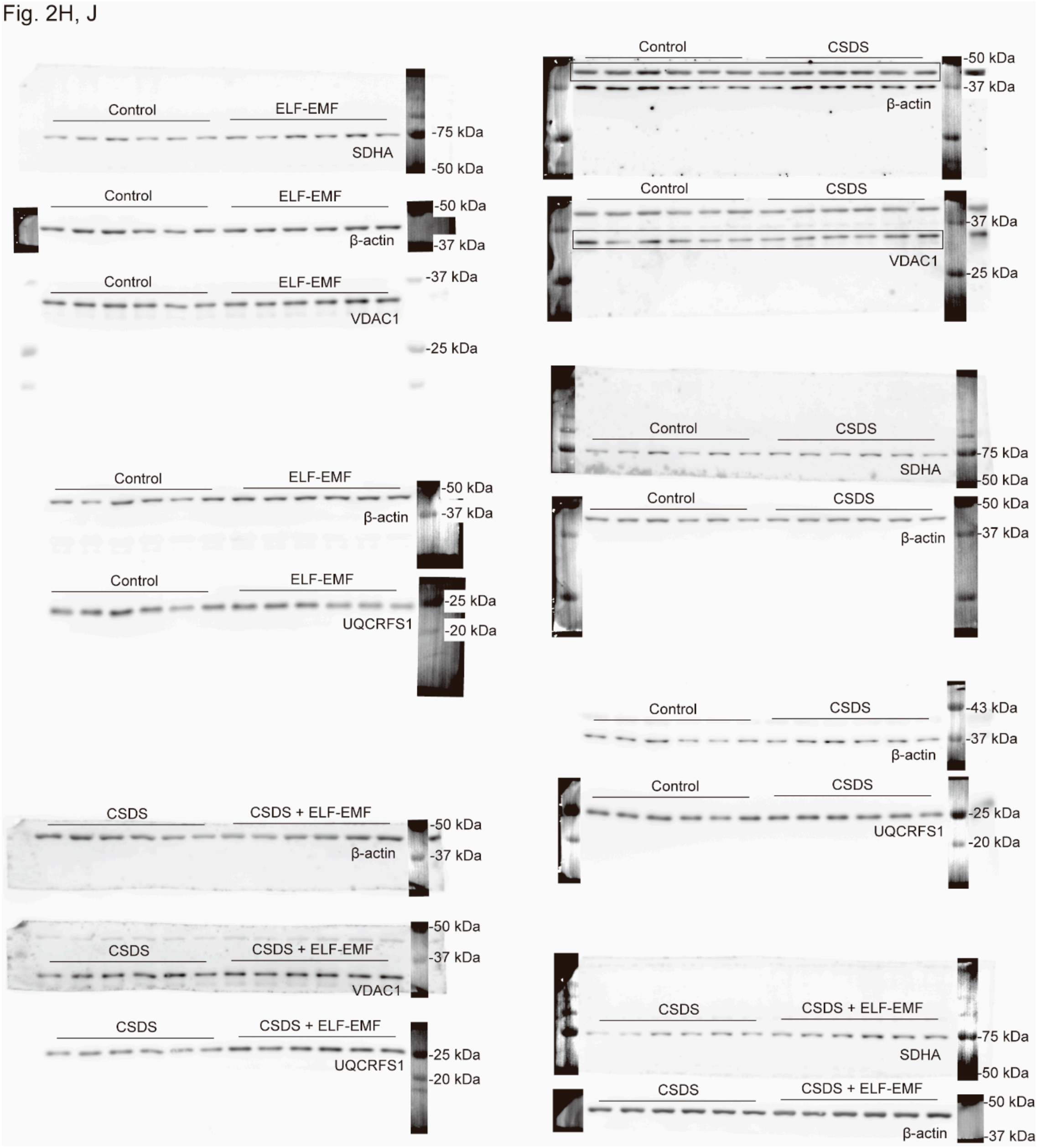

Supplementary Figure S4

Fig. 2H, J

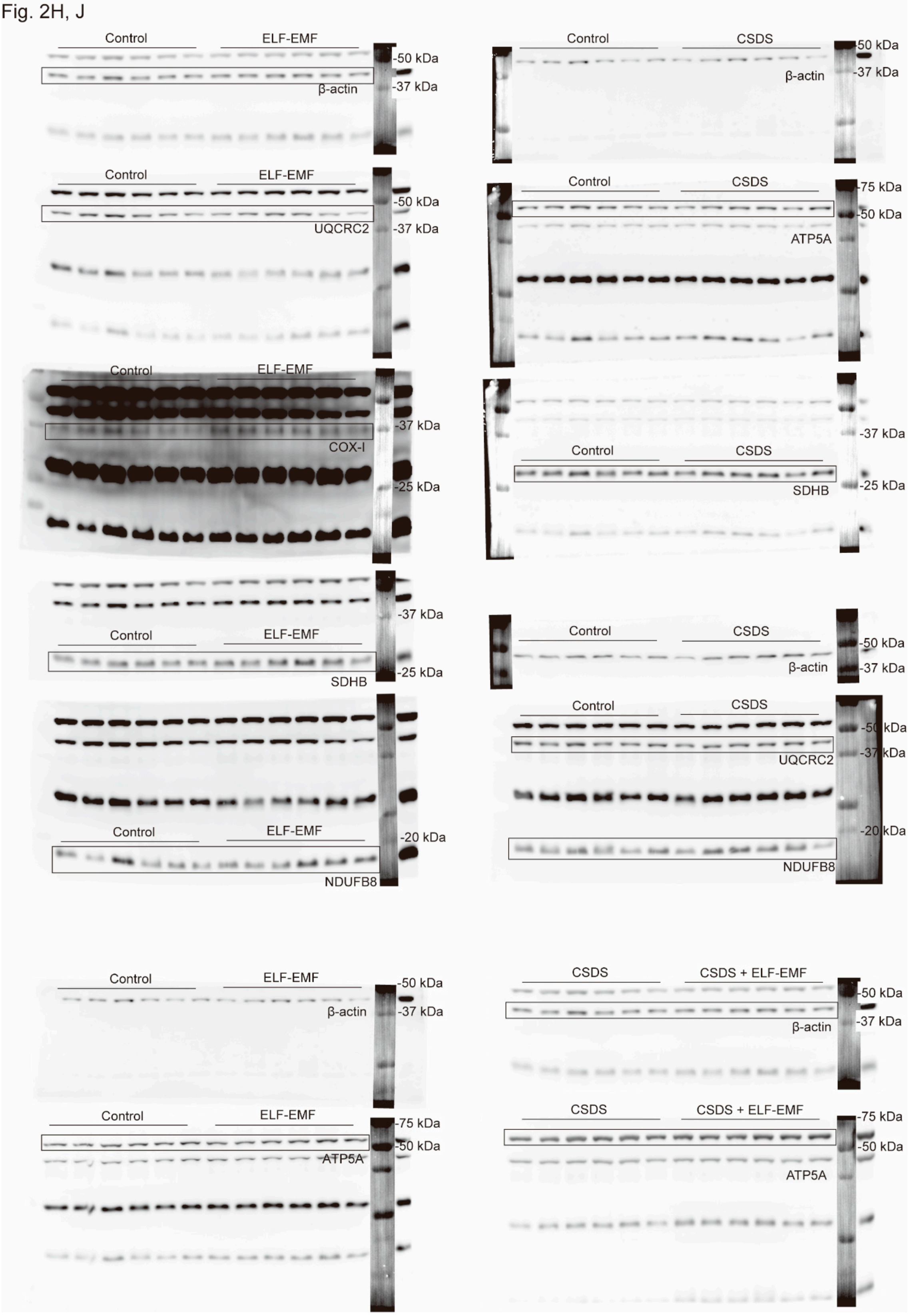

Supplementary Figure S4

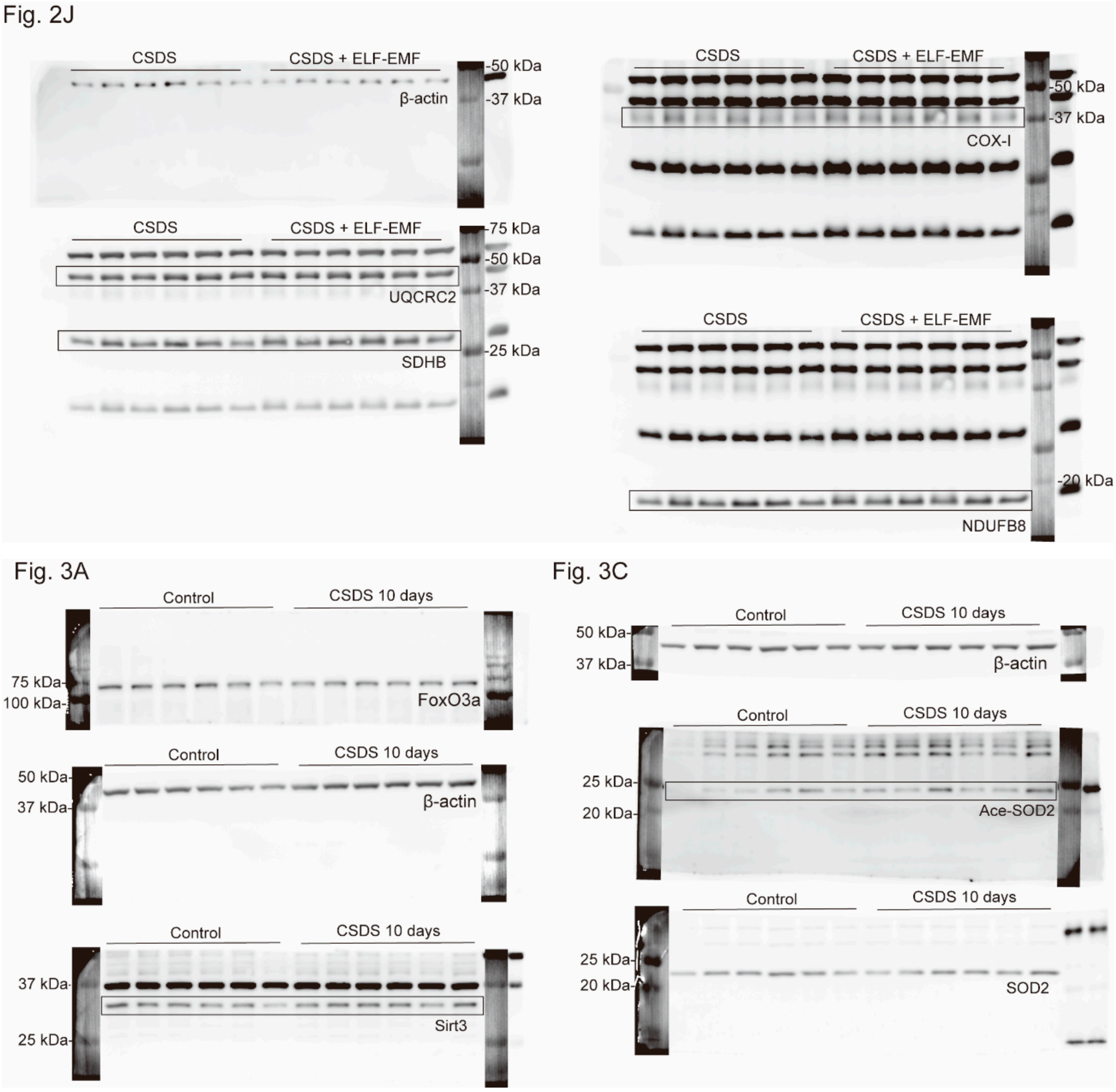

Supplementary Figure S4

Fig. 3G, I

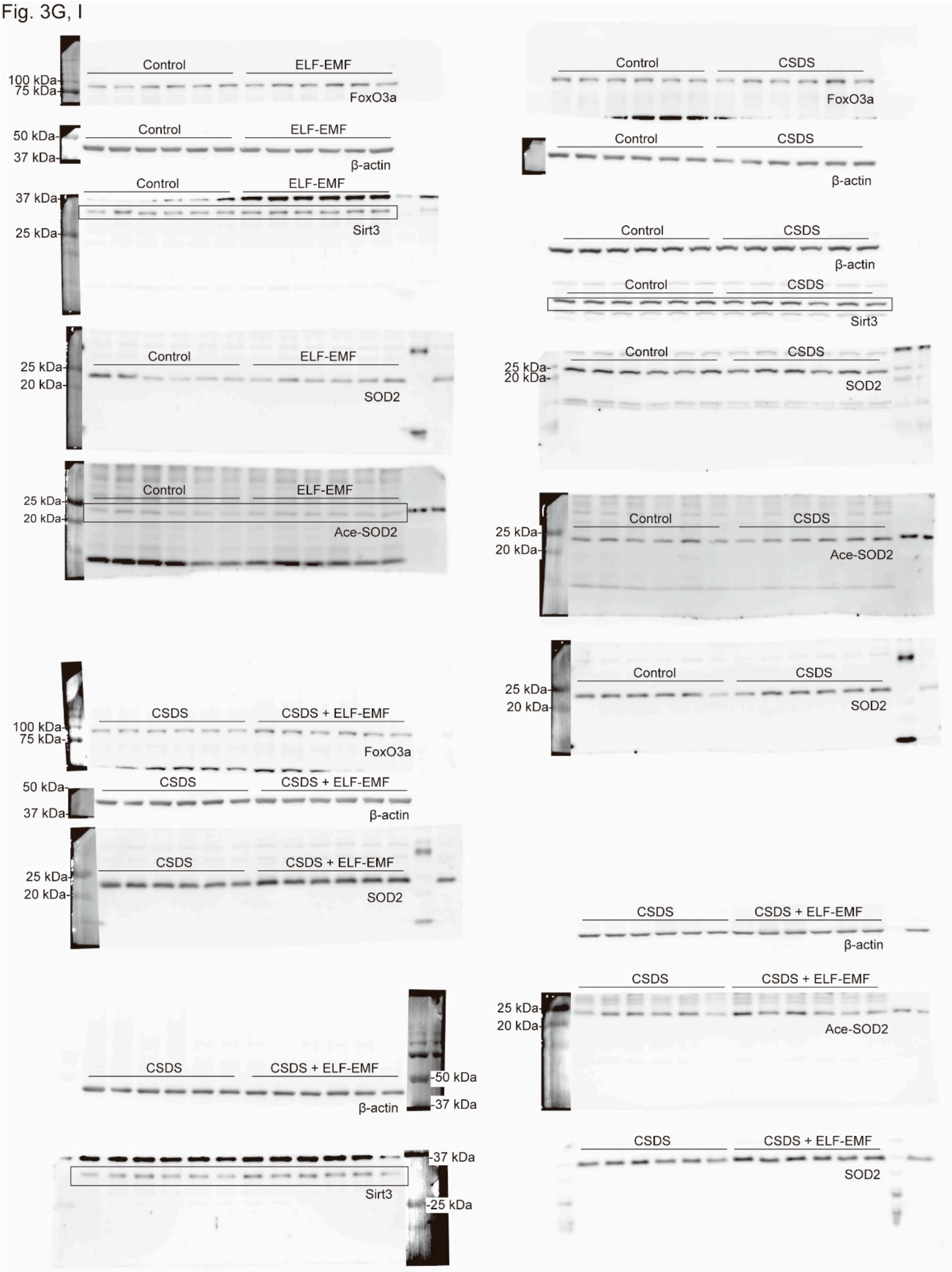

Supplementary Figure S4

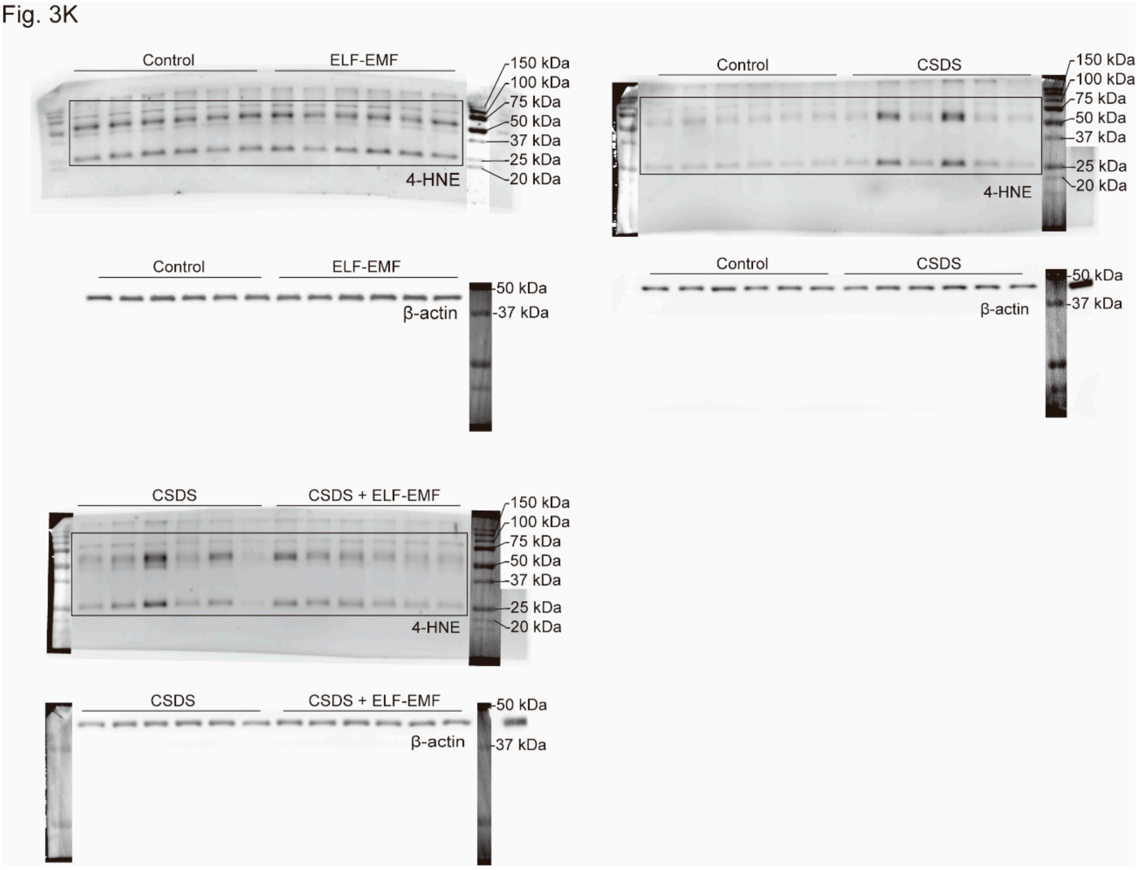

Supplementary Figure S4

Fig. 4A

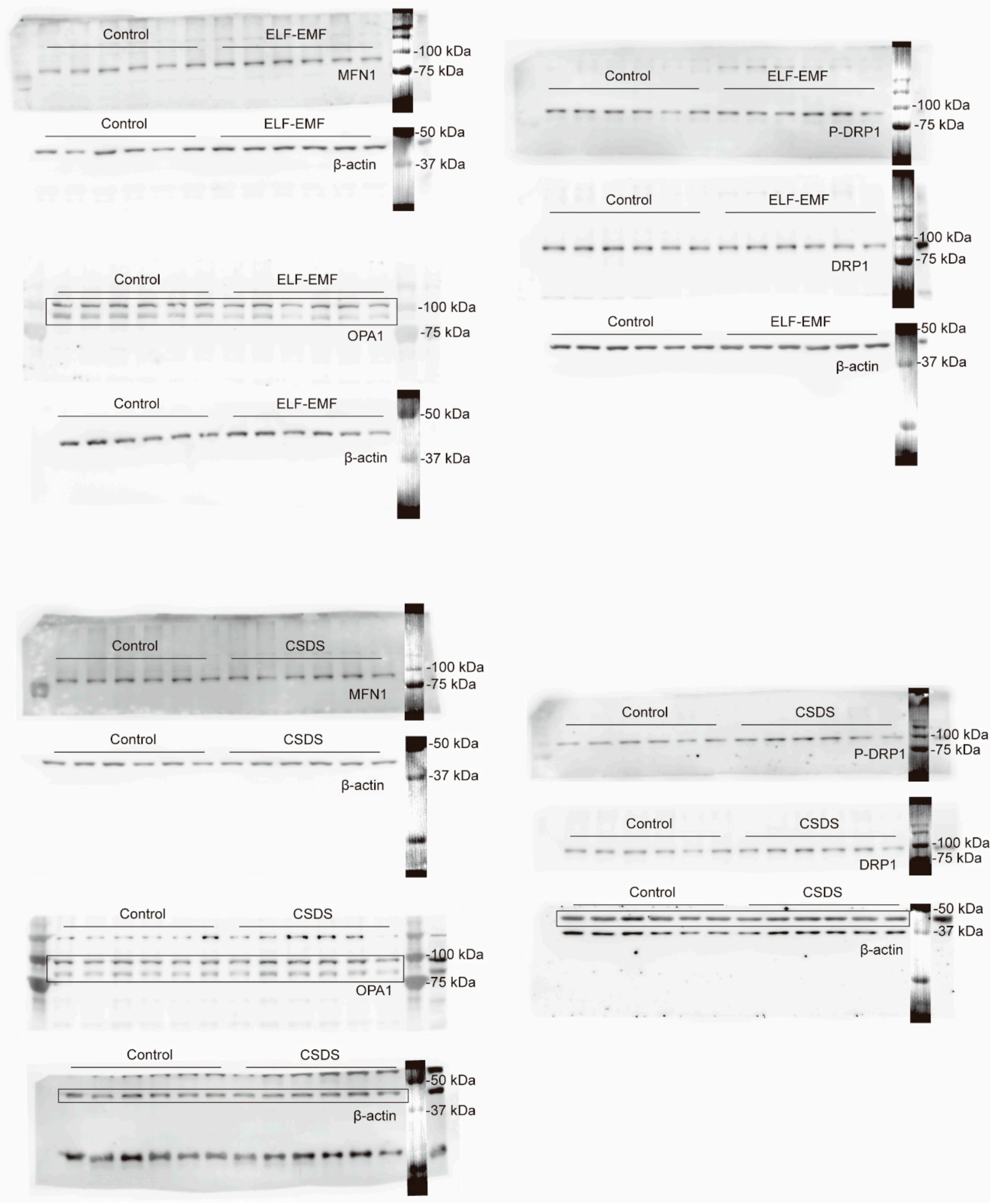

Supplementary Figure S4

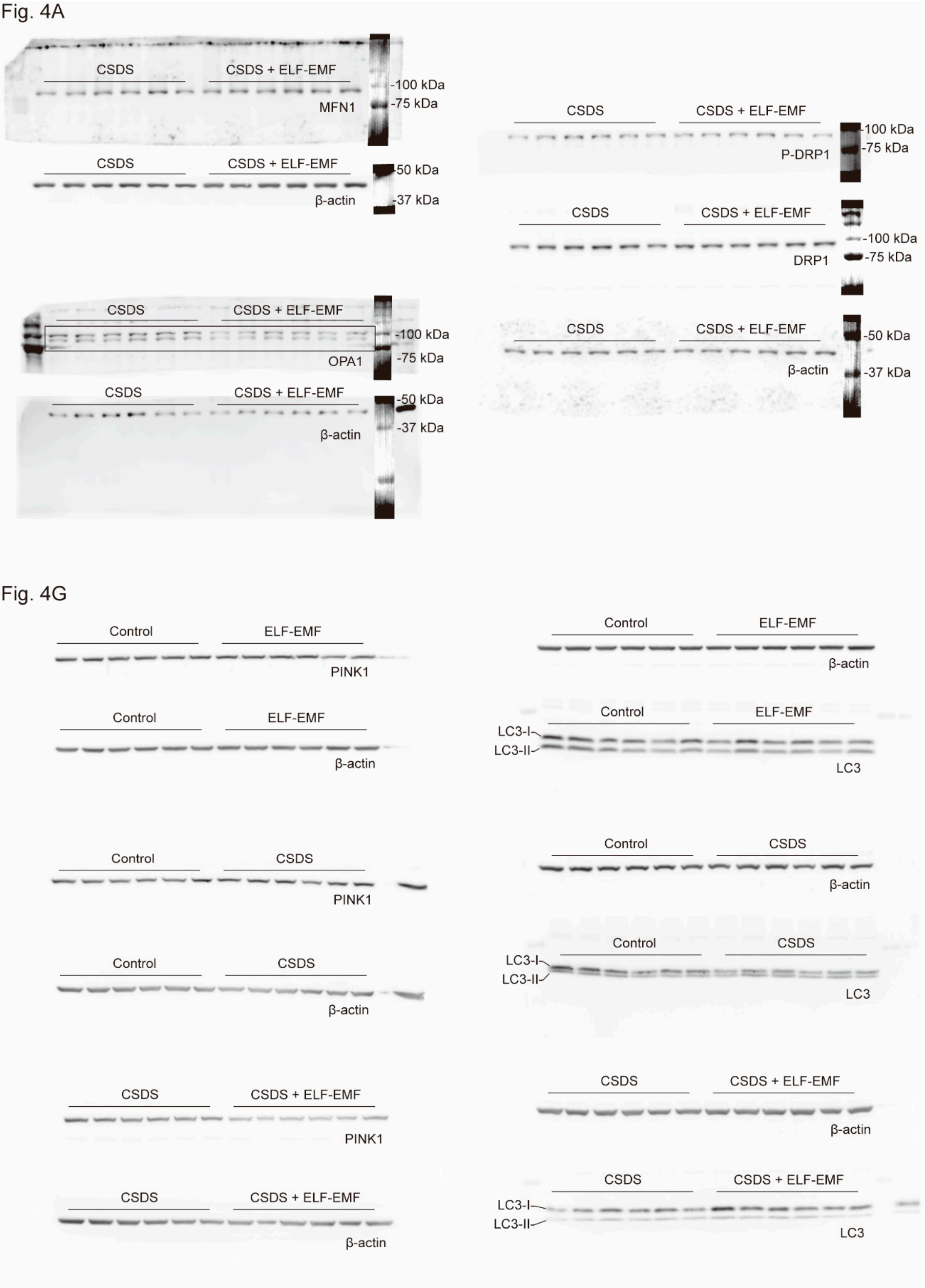

Supplementary Figure S4. Original immunoblot images for quantification
